# Notes on Preparations for the Sick

**Published:** 1887-12

**Authors:** 


### Notes on Preparations for the Sick.

---

**Lozenges**—W. T. Cooper, London.
**Jujubes**—Allen & Hanburys, London.
**Pastilles**—T. Buxton, Bristol.

We quote the following paragraph from the second edition of Mr. Lennox Browne's book on *The Throat and its Diseases*:—

"The lozenge is a convenient form for the administration of many remedies. It should be remembered that by the use of lozenges we get not only the immediate local effect, but also the constitutional action of the drug; and this is often greater in proportion than if a corresponding amount of the remedy had been taken direct into the stomach. As examples of this may be adduced Guaiacum, a comparatively small amount of which, given in the form of a lozenge, will produce constitutional symptoms: also the effervescent lozenges of Cooper, containing half a grain of calomel, one of which, taken at night, produces far more effect on bile secretion than two or three grains would if taken in the form of a pill. For the last two or three years I have administered the ingredients of Plummer's pill in the form of an effervescing lozenge, producing by this means a much quicker and more certain constitutional action, and with considerable benefit to the local condition manifested in the cases in which the drug has been indicated. The very powerful effects produced by sedative lozenges, which contain but very moderate doses of their respective anodyne ingredients, are also well known. By the use of lozenges the salivary secretion is stimulated; this fact should be borne in mind when giving astringent lozenges, which often tend to increase the dryness of the throat symptomatic of pharyngeal relaxation, unless combined with a sialagogue, as chlorate of potash.

"The British Pharmacopœia contains some formulæ for lozenges which are very useful; but a drawback to them consists in their hardness, the consequent slowness with which they dissolve, and their liability to produce erosion of the mucous membrane.

"To obviate these inconveniences the ingredients of all lozenges in the Throat Hospital Pharmacopœia have been incorporated with fruit paste, which not only renders them more palatable, but facilitates their dissolution. I have, however, found considerable gastric derangement ensue, especially in the case of children, from this form of lozenge; and to overcome this objection I have for some three years past combined the remedy with liquorice, which is demulcent and not irritating. It has, moreover, the further advantage of masking the nauseous tastes of many drugs valuable to administer in this form. The extreme saline pungency of chloride of ammonium, for example, is almost entirely removed when given in combination with liquorice."

The **Effervescing Lozenges** of William T. Cooper are now well known and much appreciated as thirst quenchers. The following is a list of other varieties of lozenge by the same manufacturer, many of which we have found to be of much value :—

**Aperient** (2 grs. jalapine).
**Astringent Voice,** made from the well-known red gum of Australia, "do not contain any irritant stimulant" (1 gr. of eucalyptus or red gum).
**Bismuth and Soda,** one after each meal (3 grs. bismuth, 5 grs. soda).
**Calomel** (½ gr.)
**Carbolic Acid** (⅙ gr.)
**Cerium, Oxalate of** (2 grs.)
**Chlorate of Potash** (3 grs.)
**Chlorate of Potash, and Eucalyptus.**
**Compound Euonymin.**
**Ipecacuanha,** an agreeable form for children ($\frac{1}{12}$ gr.)
**Iron, Phosphate of,** do not blacken the teeth (3 grs.)
**Iron and Quinine Tonic** (3 grs. iron, ¼ gr. quinine).
**Rhatany, Extract of** (2 grs.)

Messrs. Allen and Hanburys manufacture a series of **Medicated Jujubes and Pastilles,** which, having as their basis Pâte de Jujube, are soft and demulcent in themselves, whilst their rounded form and, in some cases, agreeable flavour make them usually acceptable to the patient as a vehicle for many medicinal substances. The list contains the following :—

**Morphia** ($\frac{1}{40}$ gr.)
**Ipecacuanha** (same strength as the B.P. lozenges).
**Morphia and Ipecacuanha** (gr. $\frac{1}{40}$ and gr. ¼).
**Compound Morphia and Ipecacuanha** ($\frac{1}{40}$ gr. morph., ⅙ ipec., ⅙ squill, &c.)
**Opium and Belladonna.** An agreeable substitute for the troch. opii B.P., and of the same strength.
**Aconite.** Each pastille equivalent to half a drop of the B.P. tincture.
**Compound Camphor or Voice.**
**Chlorate of Soda.**
**Lithia.** An agreeable mode of taking this alkali. Each pastille contains 1 grain.
**Benzoated Voice.** Useful to public speakers, &c. 1 or 2 may be taken shortly before any exertion of the voice.
**Chlorate of Potash.** A more agreeable form than the lozenge of the Pharmacopœia.

**Chalybeate.** (1 grain of citrate of iron.) An agreeable form of lozenge, readily taken by children.
**Rhatany.**
**Tannin.** The same strength as the troch. acid. tannic. P.B.
**Carbolic Acid.** Useful in sore throat or any condition of the stomach or mouth attended with fœtor of the breath.
**Bromide of Ammonium.**
**Chlorate of Potash and Borax.**
**Borax.**
**Chloride of Ammonium.**

Mr. Buxton has sent us specimens of a series of **Pastilles** prepared by him: they are of a firm gelatinous consistence, readily melting in the mouth at the body temperature, and are not disagreeable to the palate. His list of pastilles is as follows:

**Ext. Eucalypti et Pulv. Scillæ.**

**Am. Chlor. et Sodæ Bibor.**

**Cocain.** ($\frac{1}{5}$ gr.)

**Glycerin. et Codeia** ($\frac{1}{8}$ gr.)

**Glycerin. et Apomorphia** ($\frac{1}{32}$ gr.)

**Morphia** ($\frac{1}{36}$ gr.) **et Ipecac.** ($\frac{1}{12}$ gr.)

**Iodol.** (1 gr.)

He sends also—

**Trochisci Eucalypti Co.** (contain potass chlor., ext. eucalypti rostrati, et pulv. cubebæ.

---

**Pharmaceutical Preparations.**—W. E. Sacker, London.

Allied to the above various series of lozenges are the **Cocaine Pellets** and **Voice Discs** sent to us by Mr. Sacker. The former, each containing one-sixteenth of a grain of the alkaloid, are found to be of service as a remedy for laryngeal cough and throat irritation. The compressed Cocaine Pellets contain one-twentieth of a grain of cocaine, in combination with borax, chlorate of potash, soda, and ammonia, in a circular form. The voice discs are composed of cubebs, chlorate of potash, liquorice, and guaiacum resin.

This manufacturer also sends us a bottle of his **Syrup. Hypophosphit. Comp.**, which has the following ingredients in each fluid drachm:

Ferri hypophosphit. }

Calcis ,, } of each one grain.

Manganes. ,, }

Sodæ ,, }

Quinine, half grain.

Strychniæ, one sixty-fourth of a grain.

Preparations of this kind are now so well known as to require no detailed account of their properties.

Mr. Sacker also sends us some of the **Coated Pills** prepared by Messrs. W. H. Schieffelin & Co. These are second to none as regards "the perfection of pill-making" and efficiency of the product. Few patients will now tolerate the old-fashioned, freshly-prepared, and still moist pill, which is only prevented from sticking to its neighbours by a thick coating of magnesia or French chalk. How often have we sympathised with a patient who has been condemned to the swallowing every half-hour of a croton chloral pill—moist, sticky, and fragrant; and how often have we been prevented from prescribing this drug from a knowledge of the fact that this is the form in which it would probably be dispensed! Few patients have any difficulty in swallowing nicely-coated pills, dry, and free from taste or smell; whilst, on the other hand, the best of drugs fail in their mission when presented to the patient in a nauseous and disagreeable form. Our transatlantic pharmacists have given us the perfection of pill-making; nevertheless it is inconvenient at times to wait for our pills whilst they are being conveyed from the other side of the Western ocean. Of the numerous formulæ commonly used and in readiness, some of the most useful are the following, selected as being the most recent:

**Aloin. Strychniæ, et Belladonnæ, Pil.** (aloin, $\frac{1}{5}$ gr.; strychniæ, $\frac{1}{60}$ gr.; ext. fol. belladonnæ, $\frac{1}{8}$ gr.)

**Calcii Sulphidi, Pil.** ($\frac{1}{10}$ gr., $\frac{1}{4}$ gr., $\frac{1}{2}$ gr., 1 gr.) Dose $\frac{1}{16}$ to 1 grain. This salt, which under ordinary circumstances rapidly spoils, keeps well in the soluble coated pill.

**Cascaræ Sagradæ Ext., Pil.** (2 grs.)

**Euonymin et Coloc. Comp. Ext., Pil.** (euonymin, 1 gr.; coloc. comp. ext. P.B., 3 grs.)

**Pil Quiniæ, Ferri, et Zinci Valerianat.** (quiniæ valerianat., 1 gr.; ferri valerianat., 1 gr.; zinci valerianat., 1 gr.)

**"Ergotin" (Ext. Ergotæ), Pil.** (1 gr., 3 grs.)

**Ferruginous (Blaud's), Pil.** (ferri sulph., $2\frac{1}{2}$ grs.; potass. carb., $2\frac{1}{2}$ grs.) These pills are admirably adapted for the assimilation of iron, as the ingredients react upon each other to form protoxide of iron, which, owing to the protection afforded by the soluble coating, remains unaltered until freed for action by the stomach juices.

**Phosphori, Pil.** ($\frac{1}{100}$ gr., $\frac{1}{50}$ gr., $\frac{1}{33}$ gr., $\frac{1}{25}$ gr., $\frac{1}{20}$ gr.)

**Quiniæ, Phosphori, Ferri, et Nucis Vomicæ, Pil.** (quiniæ sulph., 1 gr.; phosphori, $\frac{1}{100}$ gr.; ferri carb.—Vallet's mass, 1 gr.; ext. nucis vomicæ, $\frac{1}{4}$ gr.)

**Ergotin Comp., Pil.** (ergotin, 3 grs.; ext. cannab. Ind., $\frac{1}{6}$ gr.; strychniæ, $\frac{1}{60}$ gr.)

It is claimed for these pills that the best materials are used in their manufacture, that they are made by the best of machinery with the greatest possible care, that they are subdivided with scrupulous exactitude, and that no volatile principles are dissipated by heat or organic matters charred.

We have found that the efficiency of therapeutic action appears to justify the claim which is made for them by their manufacturers, whose name is at once a guarantee of perfection as regards pill-making.

---

**Kronenquelle Water.**—W. Schacht & Co., London.

Ober-Salzbrunn, in Prussian Silesia, has recently obtained notoriety from the rapidly increasing popularity of the water of the Crown spring, the consumption of which had increased from 12,623 bottles in 1881 to 242,179 in 1885.

The water is clear as crystal, and perfectly colourless: it has a pleasant pungent taste, caused by small bubbles of carbonic acid. Analysis shows that it contains 2·33 grammes of saline matters per 1000, and that these consist largely of bicarbonate of soda, lime, and magnesia, with smaller portions of sulphates, and traces of lithium and iron. Its composition should give it a prominent place among the remedies calculated to prevent the deposition of urates in the joints, as well as the tendency of uric acid to crystallise within the body; and experience has shown that for these purposes it can scarcely be surpassed.

---

**Antiseptic Dressings.**—J. Milne, London.

We have received some samples of antiseptic dressings prepared by Mr. Milne with ammonio-mercuric chloride, or sal-alembroth, as it is usually called. The trustworthiness of this salt as an antiseptic is now beyond dispute, and it is extensively used. We have sometimes suspected that this salt, being cheap, has been added in excess in the dressings; at least, we have noticed pustular eruptions under some of these dressings when left unremoved on the skin for a week or longer. Milne's reputation is so well known that we can always place implicit reliance on the quality of the dressings he manufactures, not only as regards the admixture of the chemicals, but with respect to the texture and manufacture of the vehicles he employs. The gauze in dressings and bandages, and the wool in bulk and in convenient small tampons in bags, seem to be material for surgical dressings which are of the highest excellence.

---

Messrs. Parke, Davis, and Co., Detroit, U.S.A., are the proprietors of Semple's Atomizing Inhaler, which we noticed in September. Messrs. Burgoyne, Burbidges, and Co. are the agents.

